# Different impact of task switching and response-category conflict on subsequent memory

**DOI:** 10.1007/s00426-019-01274-3

**Published:** 2019-12-05

**Authors:** Michèle C. Muhmenthaler, Beat Meier

**Affiliations:** Institute of Psychology, University of Bern, Fabrikstrasse 8, 3012 Bern, Switzerland

## Abstract

The impact of cognitive control demands on long-term memory is mixed, with some conflicts leading to better, others leading to worse subsequent memory. The current study was designed to investigate how different types of cognitive control demands modulate the effects on memory. At study, participants had to switch between two classification tasks and later, free recall performance was assessed. The stimuli consisted of two interleaved words, one word had to be categorized and the other word had to be ignored. In four experiments, the congruency between target and ignored words was manipulated by changing the distractor category. This allowed us to investigate the impact of different types of conflict (i.e., task switching, perceptual load, response-category conflict, stimulus-category conflict). The results revealed that task switching impaired memory in all experiments. In Experiment 1, higher perceptual load also impaired memory. Experiments 2–4 showed that the co-activation of two words which required different responses (i.e., response-category conflict) enhanced memory performance but only when the conflict stimuli were presented in pure blocks. Overall, memory performance seems to depend on attentional policies. Withdrawing attention from target encoding results in lower memory performance. In contrast, focusing attention on the target results in enhanced memory performance.

**Electronic supplementary material:**

The online version of this article (10.1007/s00426-019-01274-3) contains supplementary material, which is available to authorized users.

## Introduction

Cognitive control enables us to regulate and coordinate thoughts and actions according to our internal goals (Braver, 2012; Posner & Snyder, 1975). Core elements of cognitive control are to protect goal-relevant behavior against distraction, to detect and resolve conflict and to update behavior in response to changing goals and circumstances (Botvinick, Braver, Barch, Carter, & Cohen, 2001; Monsell, 2003). Substantial theoretical and experimental progress has been made regarding the impact of cognitive control on immediate task performance. Larger cognitive control demands at encoding constantly slow down performance and increase the error rates (Allport, Styles, & Hsieh, 1994; Cohen, Dunbar, & McClelland, 1990; Egner, Delano, & Hirsch, 2007; Gratton, Coles, & Donchin, 1992; Mayr & Keele, 2000; Meier & Rey-Mermet, 2012; Meier, Woodward, Rey-Mermet, & Graf, 2009; Rogers & Monsell, 1995). Recently, the long-term consequences of cognitive control has been addressed, that is, the consequences on memory (Davis, Rosner, D’Angelo, MacLellan, & Milliken, 2019; Krebs, Boehler, De Belder, & Egner, 2015; Ortiz-Tudela, Milliken, Botta, LaPointe, & Lupiañez, 2017; Richter & Yeung, 2012, 2015; Rosner, D’Angelo, MacLellan, & Milliken, 2015; Rosner, Davis, & Milliken, 2015; Yue, Castel, & Bjork, 2013). Interestingly, some studies showed that larger control demands at encoding increased later memory performance while other studies showed that larger control demands at encoding decreased subsequent memory performance. In the present study, we combined different types of control demands and assessed their consequences on memory. The aim was to investigate systematically how different types of cognitive control demands affect subsequent memory performance and to explore the underlying mechanisms.

The consequences of cognitive control are usually assessed through testing subsequent memory for the stimuli that produce conflict at encoding. In a study phase, the control demands vary and in a later test phase, memory is assessed. For example, in a study by Krebs et al. (2015), participants performed a Stroop-like task. Male or female faces were overlaid with the words man, house or woman, thus congruent, neutral and incongruent face-word stimuli were created. The participants had to judge the gender of the face, while ignoring the superimposed word. The *incongruent* condition triggered a response-category conflict as the face and the distractor word required different responses. On incongruent trials, performance was slowest, but on the subsequent recognition memory test, these faces showed better memory performance, that is, a conflict-induced benefit. The authors argued that the emerging conflict in the incongruent condition served as an internal signal for reinforcing top-down attention to task-relevant information and that encoding mechanisms for incongruent targets were up-regulated which led to better memory (Botvinick et al., 2001; Egner & Hirsch, 2005).

In a related study, participants had to read one word of a pair of spatially interleaved words (Rosner, D’Angelo et al., 2015). Half of the stimuli were congruent (the words had the same identity) and the other half were incongruent (the words had different identities). Performance was slower in the incongruent condition and the results of the subsequent recognition test showed better memory performance for incongruent trials. The authors suggested that selective attention demands for incongruent stimuli cued learning processes which led to enhanced recognition memory.

Other researchers used a task-switching paradigm to investigate the impact of different control demands on memory (Chiu & Egner, 2016; Muhmenthaler & Meier, 2019; Reynolds, Donaldson, Wagner, & Braver, 2004; Richter & Yeung, 2012, 2015). In task-switching experiments, participants perform a series of simple tasks. On some trials, the task changes (switch trials), and on other trials, the task is repeated (repeat trials). For switch trials, an increase in cognitive control is necessary to reconfigure the task set (Rogers & Monsell, 1995). Moreover, as usually bivalent stimuli are used (i.e., stimuli that can be used to perform two tasks), an interference produced by overlapping stimulus features also occurs (Allport et al., 1994; Allport & Wylie, 1999; Woodward, Meier, Tipper, & Graf, 2003). Task switching is associated with performance costs (referred to as switch costs), in terms of slower task performance and increased error rates (Jersild, 1927; Meiran, 2000; Rogers & Monsell, 1995). In a study by Reynolds et al. (2004), participants had to categorize words according to two dimensions. In one condition, they had to perform one task alone (single-task block) and in another condition, they had to perform two tasks in alternating runs (task-switching block). In the task-switching block, performance was slower and less accurate and recognition memory was worse compared to the single-task block. This suggests that the requirement to switch task impaired later memory performance, that is, a memory cost induced by larger control demands.

In a more recent study, we extended this line of research in two experiments (Muhmenthaler & Meier, 2019). In Experiment 1, we used univalent materials, that is, stimuli that can only be used to perform one task. The results revealed that task switching impaired memory performance. In a second experiment with bivalent materials, this effect was even stronger, suggesting that the larger cognitive demands of bivalent compared to univalent switch trials further hurt memory encoding for task-relevant information.

Richter and Yeung (2012) investigated the impact of task switching on recognition memory for attended and unattended stimuli. They used compound stimuli which consisted of picture–word pairs and participants had to switch between classifying pictures versus words. The results showed that task switching compared to task repetition resulted in less confident recognition of the attended targets but to more confident recognition of unattended stimuli. The authors suggested that task switching impaired encoding of task-relevant information but facilitated encoding of task-irrelevant information by affecting the selectivity of memory encoding.

Together, memory performance for targets was impaired in all studies when participants had to switch task. This is in line with the assumption that the increased control demands in switch trials reduce top-down attention toward the targets (Richter & Yeung, 2015). In other words, attention is devoted to task operations which result in less-efficient target encoding and in more distractor intrusions (Lavie, Hirst, De Fockert, & Viding, 2004; Richter & Yeung, 2012).

In summary, the literature on the interplay of cognitive control at encoding and subsequent memory showed a memory benefit for incongruent compared to congruent stimuli. In contrast, there is a memory cost when participants had to switch task. In the present study, we aimed to combine these two effects to produce opposing effects on memory. Toward this goal, we used congruent and incongruent stimuli and embedded them in a task-switching procedure in the study phase. Then, we tested memory performance. Instead of a recognition test, we applied a free recall test in all experiments. Our rationale was that for free recall, more self-initiated processing is required, thus stronger effects should materialize (cf. Craik, 1986).

To anticipate the results, task switching consistently impaired memory in all experiments. However, enhancing memory with incongruent stimuli turned out to be more difficult. In Experiment 1, we used the experimental set-up by Rosner, D’Angelo et al. (2015) but found opposing effects (lower memory for incongruent stimuli). Therefore, in Experiment 2, we changed the incongruent condition from a perceptual level to the level of the response category. Nevertheless, we still found no beneficial effect for congruency in Experiment 2. In Experiment 3 (https://aspredicted.org/re78g.pdf), we blocked the incongruent and congruent stimuli to foster appropriate attentional strategies and we finally found enhanced memory for incongruent targets. In Experiment 4 (https://aspredicted.org/53si7.pdf), we tested a potential confound and excluded the possibility that the effect of Experiment 3 emerged simply due to different stimulus categories.

## Experiment 1

In Experiment 1, we combined the experimental design involving incongruency used by Rosner, D’Angelo et al. (2015) and the design used in our previous study which involved task switching (cf. Experiment 1; Muhmenthaler & Meier, 2019). In the study phase, participants had to switch between two semantic classification tasks in a predictable AABB order (cf. Rogers & Monsell, 1995). The stimuli consisted of two spatially interleaved trial-unique words (cf. Rosner, D’Angelo et al., 2015). In both tasks, participants had to categorize one of these words. Half of the stimuli were congruent (the two interleaved words were the same) and the other half were incongruent (the two interleaved words had different identities). We hypothesized that memory would be higher for incongruent stimuli compared to congruent ones, based on previous results and on the general idea that selective attention demands enhanced memory (Krebs et al., 2015; Rosner, D’Angelo et al., 2015). Moreover, we hypothesized that memory for targets shown in switch trials would be lower than memory for targets shown in repeat trials as larger control demands reduce encoding of task-relevant information (Dreisbach & Wenke, 2011; Lavie et al., 2004; Richter & Yeung, 2012).

### Method

#### Participants

The participants were 84 German-speaking volunteers from the general population (36 male and 48 female). The age ranged from 18 to 29 years (*M* = 22.23, SD 2.67). They were recruited by word of mouth and did not get any financial compensation. In an a priori power analyses (Cohen, 1988), we computed the sample size as a function of the required power level, the significance level and the population effect size which we expected (Faul, Erdfelder, Lang, & Buchner, 2007). We used the effect size for congruency (*d* = 0.65) from the study of Rosner, D’Angelo, et al. (2015) and a significance level of 0.05 and 0.90 as power level. The analysis computed a minimum of 74 participants as sample size. The study was approved by the ethical committee of the University of Bern and all participants gave written consent.

#### Materials

**Fig. 1:**
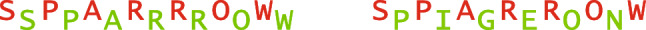
Experiment 1. Depiction of a congruent stimulus (left) involving two identical words and an incongruent stimulus (right), involving two different words from the same category

The experiment contained 36 compound word stimuli, 4 for practice and 32 for the experimental block.^1^ The compound stimuli consisted of two interleaved words which were presented in the middle of the screen as shown in Fig. 1 (cf. Milliken & Joordens, 1996; Rosner, D’Angelo, et al., 2015). One of the two words was displayed in red and the other in green against a white background in *Lucida Console* font. An experimental trial consisted either of two identical words (congruent), or two different words from the same category (incongruent). The words were exemplars of the four categories birds, mammals, music instruments and kitchen utensils and consisted of three to eight letters (cf. Muhmenthaler & Meier, 2019). For incongruent trials, the length of the words differed by a maximum of one character, the target word was always as long or longer than the distractor word. Birds and mammals were used for an animal decision task and kitchen utensils and music instruments were used for an object decision task. Word color and position were counterbalanced within each condition, so that a red target word was at the top for one half of both, congruent and incongruent trials, and at the bottom for the other half. Half of the stimuli were congruent, the other half incongruent. A total of 54 words were used (18 targets in the congruent condition, 18 targets and 18 distractors in the incongruent condition).

#### Procedure

Participants were tested in groups in a computer laboratory.

#### Study phase

**Fig. 2:**
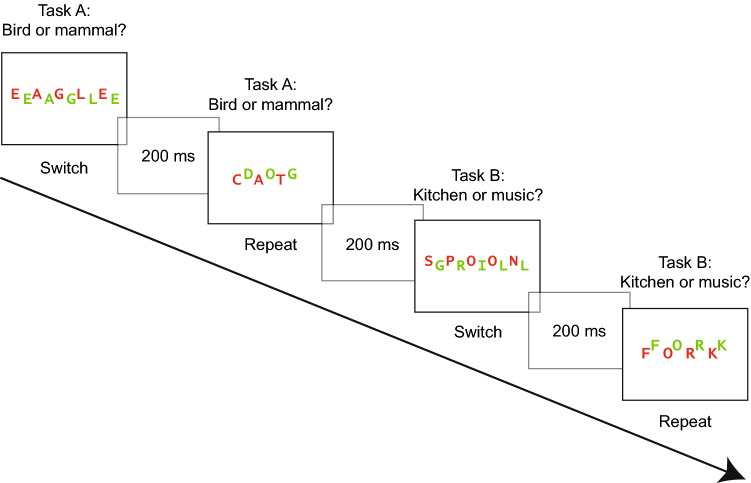
Experiment 1. Trial sequence of the study phase. Task order was a predictable AABB sequence

In the study phase, participants were informed that they will see two words on the computer screen (one red, one green) and that they will have to categorize the red word and to ignore the green word. They were instructed to switch between two classification tasks and to perform each task twice in succession. A schematic trial sequence is depicted in Fig. 2. (The words were presented in German).

In the first task, participants had to decide whether the target word was a mammal or a bird and in the second task, they had to determine if the target word was a music instrument or a kitchen utensil. They had to press the a-key for a mammal in the animal task and a music instrument in the object task and they had to respond the l-key for a bird in the animal task and a kitchen utensil in the object task. Participants were instructed to respond as quickly and correctly as possible, but they were not informed that they would be asked to recall the stimuli later. The stimuli were presented in the middle of the screen until response, in random order, with a response-stimulus interval of 200 ms. After a brief practice phase with 4 trials, participants performed the study phase with 32 trials. The stimuli were counterbalanced across participants, so that each word occurred equally often in each condition and position, and each word was presented only once to each participant. Sixteen congruent and 16 incongruent compound stimuli were presented in randomized order, counterbalanced across trial type (repeat vs. switch). Thus, each condition involved eight experimental trials.

#### Test phase

The test phase consisted of a surprise free recall test. Participants received paper and pencil, and they were instructed to write down all the words they remembered from the study phase. They had 3 min to complete this task. The entire experiment lasted about 15 min.

#### Statistical analyses

We computed the median of the reaction times (RTs) for each participant and each condition, error trials were excluded from RT analyses. Task switching performance at study was analyzed using a 2 (trial type: repeat vs. switch) × 2 (congruent vs. incongruent) analysis of variance (ANOVA) with repeated measures on correct RTs and response accuracy. Memory performance at test was analyzed with the same ANOVA. For each participant, the proportion of recalled words was computed for each condition. An alpha level of 0.05 was used for all statistical tests. Effect sizes are expressed as partial *η*^2^ values.

### Results

#### Study phase

RTs were faster for repeat (*M* = 1760 ms, SE 64 ms) than for switch trials (*M* = 2065 ms, SE 70 ms), *F*(1, 83) = 65.66, *p* < 0.001, $\eta_{\text{p}}^{2}$ = 0.44, indicating that the expected switch costs occurred. Responses were slower for incongruent (*M* = 2092 ms, SE 72 ms) than for congruent stimuli (*M* = 1733 ms, SE 63 ms), *F*(1, 83) = 75.49, *p* < 0.001, $\eta_{\text{p}}^{2}$ = 0.48. The main effect was qualified by an interaction, *F*(1, 83) = 11.05, *p* = 0.001, $\eta_{\text{p}}^{2}$ = 0.12. RTs for incongruent trials (*M* = 1859 ms, SE 73 ms for repeat, respectively, *M* = 2326 ms, SE 83 ms for switch stimuli) were stronger affected by trial type (*t*(83) = 7.17, *p* < 0.001, *d* = 0.78) than RTs for congruent trials (*M* = 1661 ms, SE 70 ms for repeat, respectively, *M* = 1804 ms, SE 64 ms for switch stimuli), *t*(83) = 2.46, *p* = 0.016, *d* = 0.27.

The same ANOVA on accuracy revealed that performance was generally high (*M* = 0.94, SE 0.01). Accuracy was significantly lower for switch (*M* = 0.91, SE 0.01) than for repeat trials (*M* = 0.97, SE 0.01), *F*(1, 83) = 44.60, *p* < 0.001, $\eta_{\text{p}}^{2}$ = 0.35. Moreover, accuracy was significantly lower for incongruent (*M* = 0.92, SE 0.01) than for congruent stimuli (*M* = 0.96, SE 0.01), *F*(1, 83) = 20.72, *p* < 0.001, $\eta_{\text{p}}^{2}$ = 0.20. The main effects were qualified by a significant interaction, *F*(1, 83) = 14.45, *p* < 0.001, $\eta_{\text{p}}^{2}$ = 0.15. In the switch condition, accuracy was substantially lower for incongruent (*M* = 0.87, SE 0.01) than for congruent trials (*M* = 0.94, SE 0.01), *t*(83) = 4.96, *p* < 0.001, *d* = 0.54, whereas in the repeat condition, accuracy was almost equal for congruent (*M* = 0.97, SE 0.01) and for incongruent trials (*M* = 0.96, SE 0.01), *t*(83) < 1, *p* = 0.333, *d* = 0.11.

#### Test phase

**Fig. 3:**
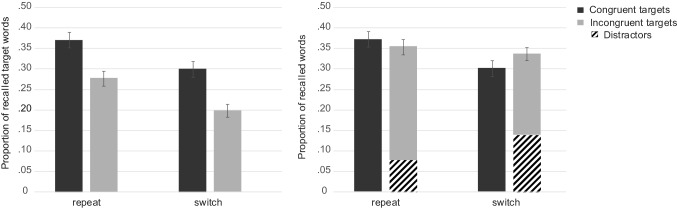
Experiment 1. Free recall performance for target words as a function of congruency modulated by trial type (left). The shaded areas reflect the distractors and the solid bars the targets (right). Error bars represent standard errors

For the test phase, overall free recall performance was *M* = 0.29 (SE 0.01), thus on average, participants correctly recalled 9.3 out of 32 target words. The average proportion of intrusions was 0.77 words (SE 0.14), however, as they cannot be assigned to any condition, we do not discuss them further. We first analyzed target memory performance with an ANOVA with the same two within-subject variables trial type (repeat, switch) and congruency (congruent, incongruent), see Fig. 3. As hypothesized, participants recalled more target words from repeat (*M* = 0.32, SE 0.01) than from switch trials (*M* = 0.25, SE 0.01) and this difference was significant, *F*(1, 83) = 32.24, *p* < 0.001, $\eta_{\text{p}}^{2}$ = 0.28. Against expectations, free recall performance for incongruent targets was significantly lower (*M* = 0.24, SE 0.01) than for congruent targets (*M *= 0.34, SE 0.01), *F*(1, 83) = 33.62, *p* < 0.001, $\eta_{\text{p}}^{2}$ = 0.29. The interaction between trial type and congruency was not significant, *F*(1, 83) < 1, *p* = 0.811, $\eta_{\text{p}}^{2}$ < 0.01.

In a second step, we also analyzed memory performance for distractors (see Fig. 3). A two-tailed paired sample *t* test revealed that significantly more distractors were recalled from the switch (*M* = 0.14, SE 0.01) than from the repeat condition (*M* = 0.08, SE 0.01), *t*(83) = 3.51, *p* = 0.001, *d* = 0.38.

### Discussion

Experiment 1 investigated whether the different control demands associated with task switching and incongruency at encoding affect subsequent free recall performance. In the study phase, participants had to switch between two semantic classification tasks, half of the stimuli were congruent (two identical words) and the other half incongruent (two different words). In the test phase, switch and incongruent stimuli impaired subsequent free recall performance. This was expected for switch stimuli as it replicated previous results (Muhmenthaler & Meier, 2019; Reynolds et al., 2004; Richter & Yeung, 2012, 2015). The enhanced control demands in switch trials withdrew attention from target processing which led to a less successful encoding for the target words. As we used univalent stimuli, the results were not influenced by stimulus bivalency as in other studies (Chiu & Egner, 2016; Richter & Yeung, 2012). Our results rather suggest that the task switching requirements affected stimulus-processing priorities (Lavie et al., 2004). This resulted in lower memory for targets but also in increased distractor encoding in switch trials, see Fig. 3 on the right side. The latter pattern replicates previous studies (Richter & Yeung, 2012, 2015).

In contrast, the finding that incongruent stimuli impaired memory was unexpected. Our experimental design was based on the study by Rosner, D’Angelo, et al. (2015). Their results showed higher memory for incongruent stimuli, but our results showed the opposite, lower memory performance for incongruent stimuli. However, a critical difference between our study and the study by Rosner et al. is that incongruency was somewhat different. In Rosner et al.’s study, participants had to read one word of a word pair in the incongruent condition, thus the target and the distractor words led to different responses, namely reading one or the other word out aloud. Therefore, a response-category conflict emerged, resulting from the co-activation of two incompatible responses (Egner et al., 2007). In contrast, in our study, participants had to categorize one of two words. As the target and the distractor in incongruent trials stemmed from the same category (e.g., two mammals in the animal task) they always required the same response. Thus, the incongruent stimuli were incongruent on a perceptual or semantic level but not response-incongruent. We reasoned that the presence of a response-category conflict may have been crucial for the memory effect in Rosner, D’Angelo et al. (2015). Accordingly, the conflict triggered by incompatible responses (reading one or the other word) led to a strategical allocation of attention toward the target word to avoid errors. In other words, participants counteracted the response-category conflict by focusing attention selectively to the target and this resulted in higher subsequent memory performance.

The same explanation can be applied to the results of Krebs et al. (2015). In their study, participants had to judge the gender of a face while ignoring the distractor word man, woman or house. An incongruent trial consisted for example of a picture of a female face and the superimposed word man, thus the target and distractor pointed to different responses. The face recognition test revealed that memory was better for incongruent stimuli compared to neutral and congruent stimuli, indicating that incongruent stimuli signaled a requirement to focus attention to the target which resulted in enhanced memory. The fact that congruent and neutral stimuli led to similar memory performance implies that incongruency in terms of a semantic mismatch in the neutral condition did not affect memory. Similarly, in the incongruent condition of our current experiment, the two words required the same response as they stemmed from the same category; therefore, focusing attention solely to the target may have been unnecessary. According to this logic, we would expect a null effect for congruency but our results revealed that even more congruent target words were recalled than incongruent ones.

A possible explanation for this result is the higher perceptual load in the incongruent condition due to the presentation of two different words instead of two identical words (Lavie, Lin, Zokaei, & Thoma, 2009). A combined analysis of all words showed that an equal amount of words from the incongruent and the congruent condition were recalled when the distractors were taken into account, as depicted in the right part of Fig. 3. This indicates that the higher perceptual load in incongruent trials rather affected the allocation of attention and not encoding per se. The presentation of two different words from the same category could have led to a spread of attention toward the distractor as this could optimize performance (Ahissar & Hochstein, 2000). This effect was more pronounced in switch trials, indicating that task switching further reduced the ability to focus on task-relevant information (Lavie et al., 2004; Richter & Yeung, 2012, 2015).

A limitation of this experiment was the different set sizes in the congruent and in the incongruent condition. By design, participants saw the same word twice on each congruent trial (as in Rosner et al.’s study). Thus, even when they attended to the green distractor word, they still encoded the correct word which could have contributed to the better memory performance for congruent targets in our study.

**Fig. 4:**
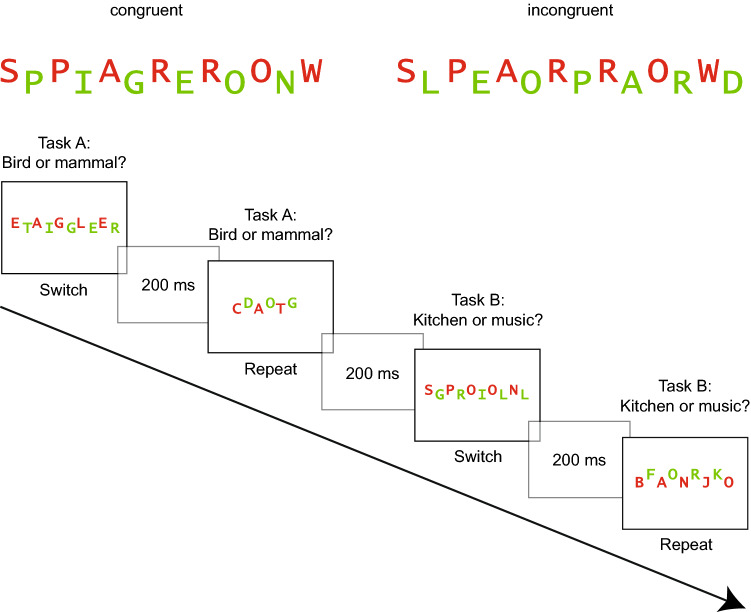
Experiment 2. Top: example of a congruent and an incongruent stimulus. Bottom: trial sequence of the study phase. Task order was a predictable AABB sequence. Congruent and incongruent stimuli were presented in randomized order

As our initial goal was to produce conflict-enhanced and switch-impaired memory, we kept the perceptual load constant in a next experiment and introduced a response-category conflict. Toward this goal, we used two words from different categories of the same task in the incongruent condition (e.g., animal task). Thus, the words required a different response (e.g., a bird requiring the *l*-key and a mammal requiring the *a*-key). In the congruent condition, we also used two different words, but from the same category, thus both words required the same response (see Fig. 4). As a consequence, the congruent condition of Experiment 2 was identical to the incongruent condition of Experiment 1.

## Experiment 2

In Experiment 2, we combined task switching with a response-category conflict. We hypothesized that memory for incongruent targets would be higher due to the possibility to counteract the response-category conflict by focusing attention to the target (Botvinick et al., 2001; Krebs et al., 2015). We further hypothesized that memory for targets in switch trials would be lower than memory for targets in repeat trials due to higher control demands in switch trials.

### Method

#### Participants

The participants were 42 undergraduate German-speaking students from the University of Bern and they participated for course credits (*M* = 22.28 years, SD 3.35, 5 male and 37 female). In an a priori power analysis (Cohen, 1988), we computed the sample size as a function of a power level of 0.90, a significance level of 0.05, and the expected joint effect size for task switching and response-category conflict of approximately *f* = 0.25 based on the observations in Experiment 1. The resulting analysis computed a number of 36 participants as an optimal sample size. As the effect for congruency was difficult to estimate, sample size considerations were also based on related studies (Krebs et al., 2015; Rosner, D’Angelo, et al., 2015). In these studies, not more than 24 participants were tested. The study was approved by the local ethical committee and all participants gave written consent.

#### Materials

The word materials were the same as in Experiment 1 but we added 18 new words. A total of 72 words were used, 18 from each category, they were used for 36 congruent and 36 incongruent stimuli. Four stimuli were used for practice and 32 for the experimental trials. In the congruent condition the two words derived from the same category within one task, thus they required the same response (e.g., two mammals). In the incongruent condition, the words derived from different categories within one task, thus they required different responses (e.g., a mammal and a bird). An example of a congruent and an incongruent stimulus is depicted in Fig. 4.

#### Procedure

The procedure was similar as in Experiment 1, with the exception that the participants were tested individually in a computer laboratory. A schematic trial sequence is depicted in Fig. 4. (Actually, the words were presented in German).

#### Statistical analyses

Data preparation was as in Experiment 1. We used a 2 (trial type: switch vs. repeat) × 2 (congruent, incongruent) ANOVA with repeated measures. For the study phase, we analyzed RTs for correctly classified stimuli and response accuracy. We excluded three participants due to accuracy below 0.70. For the test phase, the proportion of recalled words was computed for each participant and each condition. An alpha level of 0.05 was used for all statistical tests. Effect sizes are expresses as partial *η*^2^ values.

### Results

#### Study phase

RTs were faster for repeat (*M* = 1536 ms, SE 73 ms) than for switch trials (*M* = 1868 ms, SE 64 ms), *F*(1, 38) = 39.04, *p* < 0.001, $\eta_{\text{p}}^{2}$ = 0.51, indicating that the expected switch costs occurred. The RTs for incongruent (*M* = 1688 ms, SE 63 ms) and congruent trials did not differ (*M* = 1716 ms, SE 70 ms), *F*(1, 38) < 1, *p* = 0.469, $\eta_{\text{p}}^{2}$ = 0.01. The interaction between trial type and congruency was not significant *F*(1, 38) < 1, *p* = 0.865, $\eta_{\text{p}}^{2}$ < 0.01.

The same ANOVA on accuracy revealed that performance was generally high (*M* = 0.92, SE 0.01). Responses were more accurate for repeat (*M* = 0.93, SE 0.01) than for switch trials (*M* = 0.90, SE 0.01), and this difference was significant, *F*(1, 38) = 5.57, *p* = 0.023, $\eta_{\text{p}}^{2}$ = 0.13. Responses were significantly more accurate for congruent (*M* = 0.94, SE 0.01) than for incongruent trials (*M* = 0.89, SE 0.02), *F*(1, 36) = 4.89, *p* = 0.033, $\eta_{\text{p}}^{2}$ = 0.12. The interaction between trial type and congruency was not significant, *F*(1, 38) < 1, *p* = 0.821, $\eta_{\text{p}}^{2}$ < 0.01.

#### Test phase

**Fig. 5:**
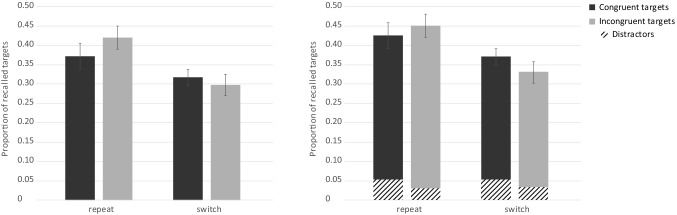
Experiment 2. Free recall performance for target words as a function of congruency modulated by trial type (left). The shaded areas reflect the distractors and the solid bars the targets (right). Error bars represent standard errors

Overall free recall performance for the targets was *M* = 0.35 (SE 0.02), thus on average, participants correctly recalled 11.2 out of 32 target words. The average proportion of intrusions was 0.68 words (SE 0.15). The detailed results are depicted in Fig. 5. An ANOVA conducted with the two same within-subject factors trial type and congruency revealed better memory for target words from repeat (*M* = 0.40, SE 0.03) than from switch trials (*M* = 0.31, SE 0.02) and this difference was significant, *F*(1, 38) = 13.07, *p* = 0.001, $\eta_{\text{p}}^{2}$ = 0.26. Free recall performance for incongruent targets was slightly better (*M* = 0.36, SE 0.02) than for congruent targets (*M* = 0.35, SE 0.03), but this difference did not reach significance, *F*(1, 38) < 1, *p* = 0.583, $\eta_{\text{p}}^{2}$ = 0.01. The interaction between trial type and congruency was not significant, *F*(1, 38) = 1.54, *p* = 0.223, $\eta_{\text{p}}^{2}$ = 0.04.

In a next step, we analyzed distractors. The ANOVA with the within-subject factors trial type and congruency revealed that a same amount of distractors were recalled from repeat (*M* = 0.04, SE 0.01) and from switch trials (*M* = 0.04, SE 0.01), *F*(1, 38) < 1, *p* = 0.881, $\eta_{\text{p}}^{2}$ < 0.01. Distractors from congruent trials (*M* = 0.05, SE 0.01) were more often recalled than distractors from incongruent trials (*M* = 0.03, SE 0.01), *F*(1, 38) = 5.89, *p* = 0.020, $\eta_{\text{p}}^{2}$ = 0.13. The interaction between trial type and congruency was not significant, *F*(1, 38) < 1, *p* = 0.910, $\eta_{\text{p}}^{2}$ < 0.01. However, due to a potential floor effect, these results must be interpreted with caution.

## Discussion

Experiment 2 investigated whether different control demands associated with task switching and response-category conflict would affect subsequent free recall performance. As in Experiment 1, participants had to switch between two tasks in a study phase in an AABB order, half of the stimuli were congruent (the two words required the same response) and the other half was incongruent (the two words required different responses). The perceptual load was kept constant, two different words were presented in both conditions. Task switching impaired performance in terms of slower reaction times and lower accuracy. The results also revealed that the subsequent free recall performance was lower when participants had to switch task: less targets from switch trials were recalled than from repeat trials, suggesting that attention was less focused on target processing under high control demands (Lavie et al., 2004; Muhmenthaler & Meier, 2019; Richter & Yeung, 2012).

Reaction times for congruent and incongruent stimuli did not differ, but an effect of accuracy emerged. Accuracy was lower for incongruent stimuli, indicating that these stimuli were perceived as more conflicting. Typically, in studies on the congruency effect, performance is also slowed on incongruent trials. However, in these studies, the number of stimuli is small and repeated (e.g., numbers), and thus automatic stimulus–response associations are established and stimulus-related variance is reduced (e.g., Kiesel, Wendt, & Peters, 2007; Meiran & Kessler, 2008). In the present study, the number of stimuli was much larger and each stimulus was only presented once. Thus, it is not possible to establish stimulus–response associations and this may explain the lack of a congruency effect at study. It is nevertheless the case that processing incongruent compared to congruent stimuli involves cognitive conflict as the target and distractor require different responses (Egner et al., 2007).

Notably, free recall performance was only numerically enhanced for incongruent compared to congruent targets. However, congruent and incongruent stimuli were presented in randomized order, that is, in a mixed block. This methodological feature may have reduced the effect of incongruency on subsequent memory. In mixed blocks, performance is usually slower compared to pure blocks, even when these blocks are incongruent (Duncan, 1977) and trial-to-trial adaptions would be necessary on about half of the trials (Los, 1994). Thus, there is no clear optimal strategy and participants may abstain from any level-specific preparation (Los, 1999). They may adopt a “worst-case scenario” which involves to prepare for the most difficult condition (Monsell, Patterson, Graham, Huges, & Milroy, 1992). They may also adopt a strategy which is viable for both conditions. Thus, it is possible that participants selectively focused on the target in both conditions, leading to similar memory performance for congruent and incongruent targets. We therefore decided to block congruent and incongruent stimuli in a follow-up experiment. We reasoned that in a pure block, focusing attention solely to the target was an optimal strategy for incongruent stimuli but not necessary for congruent stimuli.

## Experiment 3

In Experiment 3, we used a similar set-up as in Experiment 2, but we presented the congruent and incongruent stimuli in two separate blocks. We expected better memory performance for incongruent compared to congruent targets. Moreover, in line with our previous experiments, we expected lower memory performance in switch compared to repeat trials. We preregistered Experiment 3 on aspredicted.org.^2^

### Method

#### Participants

Participants were 40 German-speaking volunteers (*M* = 23.75 years, SD 5.56) (13 male and 27 female). They participated for course credits or for a financial compensation (10 CHF), they were recruited by word of mouth. Sample size was based on the same considerations as in Experiment 2. The study was approved by the local ethical committee and all participants gave written consent.

#### Materials

The materials were the same as in Experiment 2.

#### Procedure

**Fig. 6:**
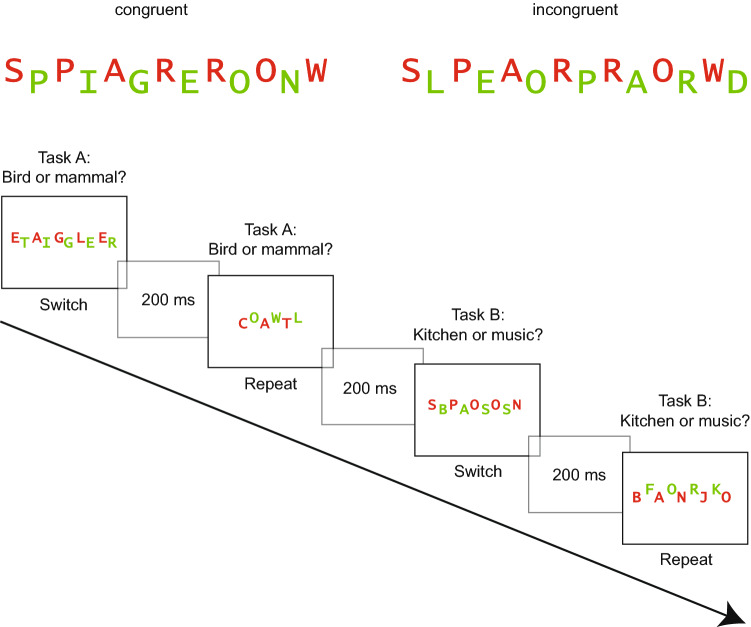
Experiment 3. Top: example of a congruent and an incongruent stimulus. Bottom: trial sequence of the study phase in the incongruent block (the congruent block is not depicted here). The task order was a predictable AABB sequence

The procedure of the study phase was the same as in Experiment 2 with the following exceptions. After a brief practice phase with 4 trials, participants performed the study phase in 2 blocks of 16 trials (see Fig. 6). The order of the blocks (congruent, incongruent) was counterbalanced. In both blocks, the instruction was the same, namely to classify the red word and to ignore the green word and to switch task after two trials. Participants were not told that the block was congruent or incongruent, respectively. After completing the task-switching procedure, participants completed an unrelated filler task to counteract potential consequences of the blocked presentation, in particular, differences in the recency effect for congruent and for incongruent stimuli. This task lasted approximately 5 min. Then, free recall was tested as in Experiments 1 and 2.

#### Statistical analyses

Data preparation was as in Experiment 1. We used a 2 (block order) × 2 (trial type: repeat vs. switch) × 2 (congruent vs. incongruent) ANOVA with repeated measures, the block order was a between-subject factor. For the study phase, we analyzed RTs for correctly classified stimuli and response accuracy. We excluded five participants with an accuracy rate below 0.70. For the test phase, the proportion of recalled words was computed for each participant and for each condition. An alpha level of 0.05 was used for all statistical tests. Effect sizes are expressed as partial *η*^2^ values. As the block order was not significant in all statistical tests, we excluded this factor from further analysis.

### Results

#### Study phase

Responses were faster for repeat (*M* = 1316 ms, SE 54 ms) than for switch trials (*M* = 1672 ms, SE 66 ms), *F*(1, 34) = 45.94, *p* < 0.001, $\eta_{\text{p}}^{2}$ = 0.58, indicating that the expected switch costs occurred. Responses were slightly slower for incongruent (*M* = 1535 ms, SE 65 ms) than for congruent trials (*M* = 1452 ms, SE 60 ms) but this difference was not significant, *F*(1, 34) = 1.35, *p* = 0.253, $\eta_{\text{p}}^{2}$ = 0.04. The interaction between trial type and congruency was not significant, *F*(1, 34) < 1, *p* = 0.547, $\eta_{\text{p}}^{2}$ = 0.01.

The same ANOVA on accuracy revealed that performance was high (*M* = 0.93, SE 0.01). Accuracy for repeat (*M* = 0.92, SE 0.02) and switch trials (*M* = 0.94, SE 0.01) did not significantly differ, *F*(1, 34) < 1, *p* < 0.444, $\eta_{\text{p}}^{2}$ = 0.02. The accuracy did also not differ for incongruent (*M* = 0.93, SE 0.02) and congruent trials (*M* = 0.94, SE 0.01), *F*(1, 34) < 1, *p* = 0.601, $\eta_{\text{p}}^{2}$ = 0.01, although it was slightly worse for incongruent trials. The interaction between trial type and congruency was not significant, *F*(1, 34) < 1, *p* = 0.898, $\eta_{\text{p}}^{2}$ < 0.01.

##### Test phase

**Fig. 7:**
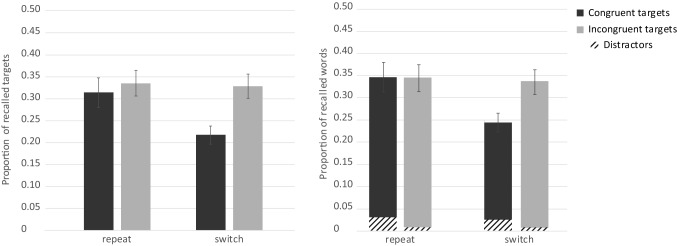
Experiment 3. Free recall performance for target words as a function of congruency modulated by trial type (left). The shaded areas reflect the distractors and the solid bars the targets (right). Error bars represent standard errors

Overall free recall performance for the targets was *M* = 0.30 (SE 0.02), thus on average, participants correctly recalled 10.2 of a total of 32 target words. The average proportion of intrusions was 1.46 words (SE 0.23). The detailed results are depicted in Fig. 7. An ANOVA with the within-subject factors trial type and congruency revealed that the participants recalled more words from repeat (*M* = 0.33, SE 0.02) than from switch trials (*M* = 0.27, SE 0.02) and this difference reached significance, *F*(1, 34) = 5.14, *p* = 0.030, $\eta_{\text{p}}^{2}$ = 0.13. Critically, free recall performance for incongruent targets was significantly better (*M* = 0.33, SE 0.02) than for congruent targets (*M *= 0.27, SE 0.02), *F*(1, 34) = 4.38, *p* = 0.044, $\eta_{\text{p}}^{2}$ = 0.11. The interaction between trial type and congruency was not significant, *F*(1, 34) = 1.82, *p* = 0.186, $\eta_{\text{p}}^{2}$ = 0.05.

In a next step, we analyzed distractors. An ANOVA with the within-subject factors trial type and congruency revealed that recall did not differ between repeat and switch trials (*M* = 0.02, SE 0.01), *F*(1, 34) < 1, *p* = 0.786, $\eta_{\text{p}}^{2}$ < 0.01. However, distractors from congruent trials (*M* = 0.03, SE 0.01) were more often recalled than distractors from incongruent trials (*M* = 0.01, SE 0.01), *F*(1, 34) = 4.60, *p* = 0.039, $\eta_{\text{p}}^{2}$ = 0.12. The interaction between trial type and congruency was not significant, *F*(1, 34) < 1, *p* = 0.812, $\eta_{\text{p}}^{2}$ < 0.01.

#### Manipulation check

To check whether performance was indeed slower in the mixed block compared to the pure blocks, we compared RTs of the study phases from Experiment 2 and 3 with a 2 (congruent vs. incongruent) × 2 (Experiment 2 vs. Experiment 3) mixed ANOVA. Repeat and switch conditions were collapsed. RTs in Experiment 3 (*M* = 1494, SE 60) were significantly faster than in Experiment 2 (*M* = 1702, SE 57), *F*(1, 72) = 6.31, *p* = 0.014, $\eta_{\text{p}}^{2}$ = 0.08. All other effects were not significant, *F*(1, 72) < 2.00, *p* > 0.163, $\eta_{\text{p}}^{2}$ < 0.03, indicating that the blocked presentation had the expected effect.

### Discussion

The aim of Experiment 3 was to investigate the impact of a response-category conflict and task switching on free recall performance. We presented congruent and incongruent stimuli in two separate blocks instead of one mixed block as in Experiment 2. The blocked presentation indeed reduced the control demands as revealed in the manipulation check. This suggests that the blocked presentation of congruent and incongruent stimuli was easier to perform and that resources were available to apply appropriate attentional strategies. Perceptual load was kept constant (two different words were presented in each trial).

Task switching slowed down performance at study and impaired subsequent free recall performance, indicating that it withdrew attention from target encoding. Critically, in Experiment 3, memory performance for incongruent targets was significantly higher than for congruent targets. Thus, the effect of a response-category conflict unfolded due to the opportunity to adopt an appropriate attentional strategy (i.e., focus on targets in the incongruent condition and relaxed focus in the congruent condition) and as a consequence, memory for incongruent target words was enhanced.

Nevertheless, an alternative explanation would be that rather than response-category conflict, the stimulus-category conflict (the co-activation of two different categories) was critical. Specifically, in the congruent condition, both target and distractor were from the same stimulus category and required the same response (e.g., *cat* and *dog*). In contrast, in the incongruent condition, the target and the distractor were from different stimulus categories (e.g. eagle is a bird and tiger a mammal) and they required a different response (i.e., a *l*-key vs. *a*-key response). Therefore, response-category and stimulus-category conflict may be confounded. To test this possibility, in Experiment 4, we kept the response-category conflict constant but varied the stimulus-category conflict by presenting two different words from different categories in the incongruent condition which required the same response (e.g., eagle is a bird and spoon is a kitchen utensil, and both require a *l*-response). If the response-category conflict is indeed critical, then memory performance should not be affected by the stimulus-category conflict. In contrast, if the stimulus-category effect is critical the same memory effect should occur as in Experiment 3.

## Experiment 4

Experiment 4 was designed to test whether the presence of a response-category conflict was critical for the memory benefit in Experiment 3 and to rule out the possibility that these results were based on a stimulus-category conflict (i.e., the co-activation of different categories) which was also present in incongruent trials. Experiment 4 was preregistered on *aspredicted.org*.^3^

### Method

#### Participants

The participants were 40 undergraduate German-speaking students from the University of Bern and they participated for course credits (*M* = 22.76 years, SD 2.89, eight male and 32 female). Sample size was based on the same considerations as in Experiments 2 and 3. The study was approved by the local ethical committee and all participants gave written consent.

#### Materials

**Fig. 8:**
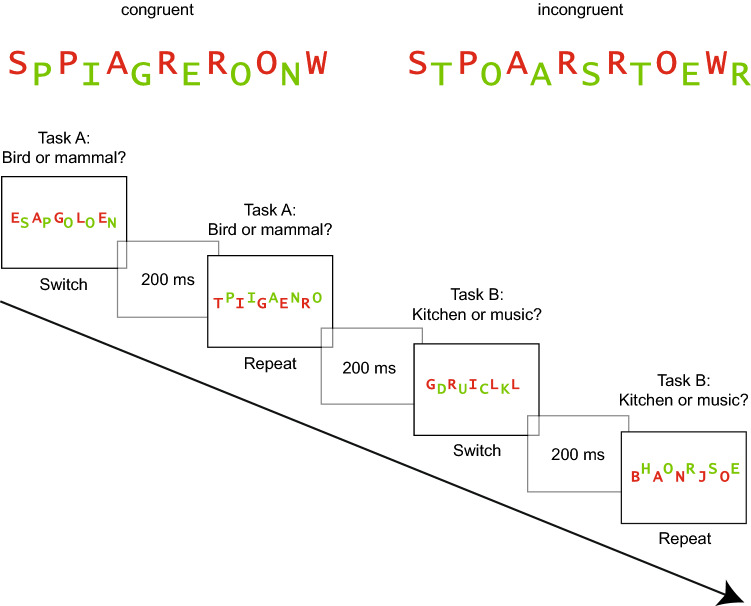
Experiment 4. Top: example of a congruent and an incongruent stimulus. Bottom: trial sequence of the study phase in the incongruent block (the congruent block is not depicted here). Both words of a trial required the same responses. The task order was a predictable AABB sequence

The word materials were the same as in Experiment 2 and 3 but they were differently combined. In the congruent condition, the two words stemmed from the same category. In the incongruent condition, the words stemmed from different categories but they required the same response, thus a pure stimulus-category conflict was induced. Birds were combined with kitchen utensils (both requiring the *l*-key) and mammals were combined with music instruments (both requiring the *a*-key). An example is presented in Fig. 8. A total of 72 words were used, 18 from each category. They were used for 36 congruent and 36 incongruent stimuli. Four stimuli were used for practice and 32 for the experimental trials.

#### Procedure

The procedure was the same as in Experiment 3. Congruent and incongruent stimuli were presented in two separate task-switching blocks. A possible trial sequence is depicted in Fig. 8.

#### Statistical analysis

Data preparation was as in Experiment 3. We used a 2 (block order) × 2 (trial type: repeat vs. switch) × 2 (congruent vs. incongruent) ANOVA with repeated measures, the block order was a between-subject factor.

### Results

#### Study phase

Means of the RTs were faster for repeat (*M* = 1550 ms, SE 88 ms) than for switch trials (*M* = 1818 ms, SE 74 ms), *F*(1, 39) = 22.97, *p* < 0.001, $\eta_{\text{p}}^{2}$ = 0.37, indicating that the expected switch costs occurred. RTs for incongruent (*M* = 1711 ms, SE 82 ms) and congruent stimuli (*M* = 1658 ms, SE 77 ms) did not differ significantly, *F*(1, 39) = 1.39, *p* = 0.246, $\eta_{\text{p}}^{2}$ = 0.03. The interaction between trial type and congruency was not significant, *F*(1, 39) < 1, *p* = 0.783, $\eta_{\text{p}}^{2}$ < 0.01.

The analysis of accuracy revealed a main effect of trial type, performance was more accurate for repeat (*M* = 0.93 ms, SE 0.01 ms) than for switch trials (*M* = 0.90 ms, SE 0.01 ms), *F*(1, 39) = 5.48, *p* = 0.024, $\eta_{\text{p}}^{2}$ = 0.12. Accuracy for congruent stimuli (*M* = 0.90 ms, SE 0.01 ms) was lower than for incongruent stimuli (*M* = 0.93 ms, SE 0.01 ms) but this difference was not significant, *F*(1, 39) = 3.66, *p* = 0.063, $\eta_{\text{p}}^{2}$ = 0.09. The interaction between trial type and congruency was not significant, *F*(1, 39) = 3.47, *p* = 0.070, $\eta_{\text{p}}^{2}$ = 0.08.

#### Test phase

**Fig. 9:**
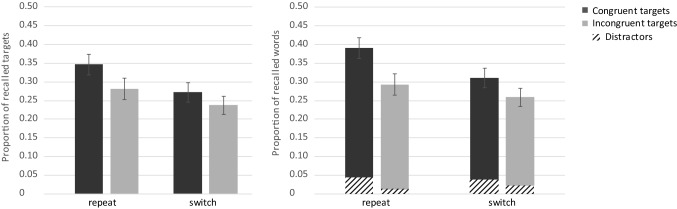
Experiment 4. Free recall performance for target words as a function of congruency modulated by trial type (left). The shaded areas reflect the distractors and the solid bars the targets (right). Error bars represent standard errors

For the test phase, overall free recall performance was *M* = 0.28 (SE 0.02), thus on average, participants correctly recalled 9.2 out of 32 target words. The average proportion of intrusions was 1.65 words (SE 0.32). We analyzed target memory performance with an ANOVA with the same two within-subject variables, trial type and congruency. The results are depicted in Fig. 9. Participants recalled more target words from repeat (*M* = 0.32, SE 0.03) than from switch trials (*M* = 0.26, SE 0.02) and this difference was significant, *F*(1, 39) = 4.66, *p* = 0.037, $\eta_{\text{p}}^{2}$ = 0.11. As hypothesized, free recall performance for incongruent (*M* = 0.26, SE 0.02) and congruent targets (*M* = 0.31, SE 0.02) did not differ significantly, *F*(1, 39) = 2.49, *p* = 0.122, $\eta_{\text{p}}^{2}$ = 0.06. The interaction between trial type and congruency was not significant, *F*(1, 39) < 1, *p* = 0.415, $\eta_{\text{p}}^{2}$ = 0.02.

The null effect of stimulus-category congruency on memory performance indicates that in Experiment 3, response-category conflict was the cause of the memory effect. Notably, in Experiment 4, numerically, memory was even better for congruent than for incongruent targets. However, as traditional statistics are not suitable to confirm null effects, a Bayesian analysis was conducted (Wagenmakers et al., 2015). The Bayes factor (BF) represents a ratio between the likelihood of two hypotheses. A BF of above 3 indicates evidence for the alternative hypothesis and below 1/3 evidence for the null hypothesis, whereas values ranging from 1/3 to 3 are indicators of inconclusive evidence (Dienes, Coulton, & Heather, 2018). Using JASP (Version 0.8.6), we calculated a Bayesian one-sided paired sample *t* test on congruency. The null hypothesis represents the possibility that congruent and incongruent targets are equally often recalled. The alternative hypothesis states that incongruent targets are more often recalled than congruent targets. The resulting BF of 0.071 indicates strong evidence for the null hypothesis (i.e., is 14 times more likely than the alternative hypothesis; Jarosz & Wiley, 2014). Accordingly, memory performance for congruent and incongruent targets does not differ and we conclude that the results of Experiment 3 are not confounded by stimulus-category conflict.

In a next step, we analyzed distractors. The ANOVA with the within-subject factors trial type and congruency revealed that a same amount of distractors were recalled from repeat and switch trials (*M* = 0.03, SE 0.01), *F*(1, 39) < 1, *p* = 0.881, $\eta_{\text{p}}^{2}$ < 0.01. Distractors from congruent trials (*M* = 0.04, SE 0.01) were more often recalled than distractors from incongruent trials (*M* = 0.02, SE 0.01), *F*(1, 39) = 6.57, *p* = 0.014, $\eta_{\text{p}}^{2}$ = 0.14. The interaction between trial type and congruency was not significant, *F*(1, 39) < 1, *p* = 0.405, $\eta_{\text{p}}^{2}$ = 0.02. Note, however, that the performance was close to floor and the results of the distractor analysis have to be interpreted cautiously.

### Discussion

Experiment 4 was designed to test the assumption that the response-category conflict was critical for a memory benefit in Experiment 3 and not the stimulus-category conflict which was also present in the incongruent condition. To disentangle these possibilities, we varied the stimulus categories in Experiment 4 and eliminated the response-category conflict. In the congruent condition, both words of the stimulus stemmed from the same category, whereas in the incongruent condition, both words stemmed from different categories but required the same response. We hypothesized that both conditions should lead to similar memory performance as no response-category conflict emerged. Moreover, we expected switch-impaired target memory.

The results revealed switch costs in terms of slower task performance and lower accuracy. Again, free recall performance was enhanced for repeat compared to switch trials. However, free recall performance for congruent and incongruent did not differ, indicating that the stimulus-category conflict did not affect memory performance. Thus, co-activation of two incompatible response alternatives was crucial for the memory improvement in Experiment 3. The presence of a response-category conflict signaled a requirement to focus attention solely to the target in order to produce a response that was not conflicting. This resulted in increased attention toward the target which later improved long-term memory. In contrast, in the experiment presented here, such a requirement was not signaled by the incongruent stimuli as target and distractor required the same response.

## General discussion

**Fig. 10:**
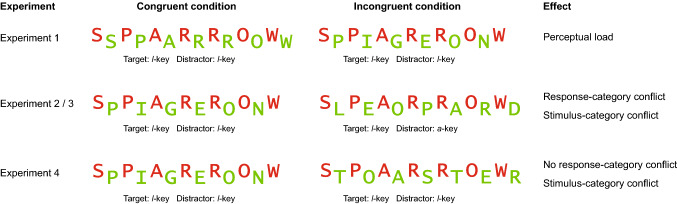
Depiction of all stimulus conditions with the assigned response keys. Note that congruent and incongruent stimuli were presented in pure blocks in Experiments 3 and 4

The impact of different types of control demands on memory was investigated in four experiments. At study, we combined a task-switching procedure with different types of incongruent conditions. An overview of all experimental conditions is presented in Fig. 10. In Experiment 1, the control demands were enhanced due to a higher perceptual load. In Experiments 2 and 3, the conflict arose from the co-activation of two words which required different responses (i.e., involved a response-category conflict). In Experiment 4, the conflict arose from the co-activation of two words from different categories (i.e., involved a stimulus-category conflict). The study integrates results from previous studies which showed that different types of control demands can have opposing effects on recognition memory (Krebs et al., 2015; Muhmenthaler & Meier, 2019; Richter & Yeung, 2012, 2015; Rosner, D’Angelo, et al., 2015, Rosner, Davis, et al. 2015; Yue et al., 2013). It also extends the generality of these results using free recall as memory measure. Free recall requires more effortful processing than recognition. More self-initiated processing is involved as no retrieval cues are available and the participants have to initiate appropriate operations more effortful (Craik, 1986). We, therefore, expected stronger effects. Moreover, in the studies by Krebs and colleagues and Richter and Yeung, “remember” responses turned out to be more sensitive than “know” responses for these types of manipulations. As “remember” responses reflect recollection which is similar (albeit not identical) to free recall (Yonelinas, 2002), we reasoned that free recall might represent a more sensitive measure. In the following sections, the results are discussed by type of conflict.

### Implications by conflict type

#### Perceptual load

In Experiment 1, the incongruent condition consisted of two different words compared to two identical words in the congruent condition. Therefore, the perceptual load was enhanced (Lavie et al., 2009) and this impaired subsequent free recall performance for the target words. It seemed that attention was distracted when the perceptual load was high, which reduced target encoding but facilitated encoding of the unattended distractors. In line with the idea that distractor processing could optimize performance, the results may also rely on a strategical spread of attention to the distractors in the incongruent condition (Ahissar & Hochstein, 2000). However, this is only one interpretation. A limitation was that there was a difference in set size. The words in the congruent condition were presented twice which might have facilitated memory encoding.

#### Response-category conflict

A response-category involves the co-activation of two incompatible representations which require different responses (Egner et al., 2007). According to the conflict-monitoring model (Botvinick et al., 2001), detecting a conflict serves as an internal signal for reinforcing top-down attention to task-relevant information. Egner and Hirsch (2005) showed that cognitive control mechanisms amplify cortical responses to task-relevant information in response to a response-category conflict. This conflict-driven attentional enhancement leads to higher target memory. In Experiment 3, we replicated previous studies that found enhanced memory performance for incongruent compared to congruent targets (Krebs et al., 2015; Rosner, D’Angelo, et al., 2015). Behaviorally, we assume that attention was strategically focused to the targets when a response-category conflict was present. The incongruent stimuli signaled a requirement to focus attention solely to the target to produce a response that was not conflicting. This is in line with the conflict-monitoring model which suggests that conflict-driven attentional enhancement toward a target leads to successful encoding (Botvinick et al., 2001).

However, enhancing memory by inducing a response-category conflict is not easily achieved. Our experiments revealed that this benefit on memory is reduced when the participants cannot adopt appropriate processing strategies. For example, the mixed presentation of congruent and incongruent trials resulted in a strategy which was viable for both conditions, hence no effect on memory emerged. In a recent study, Ptok, Thomson, Humphreys, and Watter (2019) investigated a response-category conflict in a semantic priming paradigm. Similar to our study, they found a beneficial effect on memory only under specific circumstances. They showed that only tasks that lead to automatic processing result in a beneficial effect on memory, while attention-demanding evaluative processing can eliminate the effect. They concluded that the response-category conflict must elicit control processes which directly focus on the core meaning of the target stimuli. Together, these and our results showed that subtle processing manipulations can influence the effect of a response-category conflict on memory.

Previous research showed that the effect of response-category conflict emerged despite using different tasks at study, namely word reading in Rosner, D’Angelo, et al. (2015), a face-word Stroop task in Krebs et al. (2015), semantic priming in Ptok et al. (2019) and word categorization in our study. This indicates that the effect of response-category conflict is quite robust across different paradigms. In the present study, the effect emerged in combination with task switching. We assume that in an experimental set-up with only one task to perform, stronger effects would materialize. Further research is necessary to specify boundary conditions for this type of conflict.

#### Stimulus-category conflict

A stimulus-category conflict is defined as the co-activation of two different stimulus categories. This type of conflict was present in the incongruent conditions of the Experiments 2–4. However, in the Experiments 2 and 3, it was confounded with a response-category conflict in the incongruent condition. Therefore, in Experiment 4, we tested the pure effect of a stimulus-category conflict. The results clearly showed that there was a null effect of stimulus-category conflict on memory, in line with the assumption that focusing on the target was of no avail when target and distractor required the same response.

Regarding memory performance for distractors, we found a similar pattern in Experiments 2, 3 and 4. Memory for incongruent distractor words was always lower than memory for congruent distractor words. This can be explained by a strategical spread of attention toward the distractor in the congruent condition at study. As target and distractor stemmed from the same category, spreading attention toward the distractor could help to optimize performance. However, this finding might also be explained by more memory intrusion from the same category (i.e. congruent distractors) than from a different category (i.e. incongruent distractors) at retrieval by spreading activation throughout the semantic network (Anderson, 1983). Thus, the difference in recalled distractors might reflect a retrieval effect and not necessarily an encoding effect. To decide between these possibilities, further research is necessary.

#### Task switching

All experiments replicated that task switching impairs encoding of task-relevant information (Muhmenthaler & Meier, 2019; Reynolds et al., 2004; Richter & Yeung, 2012, 2015). Task switching withdraws attention from target encoding in order to enable operations on task level. This also allows distractor intrusion by affecting stimulus-processing priorities (Lavie et al., 2004; Richter & Yeung, 2012, 2015), see Experiment 1, Fig. 3. In other words, performance is less shielded against distraction when participants have to switch task (Dreisbach & Wenke, 2011), and this results in less successful target encoding. Richter and Yeung (2015) manipulated the control demands in a task-switching procedure by varying the preparation time, by voluntary and involuntary switching and by rewards. The results revealed that these manipulations led to efficient top-down control, resulting in enhanced target memory and less distractor intrusions. Together, larger control demands as produced by task switching or other control manipulations reduce encoding of task-relevant information but they facilitate encoding of task-irrelevant information. That is, they lead to a “broadening” of cognitive control.

## Conclusion

The main goal of the study was to produce control-enhanced and control-impaired target memory within the same experiment. Task switching was combined with different types of congruency manipulations. We present the first evidence for switch-impaired and conflict-enhanced memory performance within one experiment. While task switching consistently impaired target memory in all experiments, response-category conflict had a weaker effect and it emerged only when appropriate strategies could be applied. Stimulus-category conflict did not affect memory, indicating that the co-activation of two response alternatives is critical for a memory benefit (Botvinick et al., 2001; Egner et al., 2007).

Opposing effects on memory reflect that the allocation of attention at study is crucial for later memory performance. Attention toward the targets is impaired when the control demands are enhanced due task switching requirements or due to a high perceptual load. These conditions withdraw attention from target processing, resulting in decreased target memory but also in enhanced distractor encoding. In contrast, the presence of a response-category conflict leads to focused attention toward the target resulting in increased target memory. Cognitive control mechanisms seem to amplify cortical responses to task-relevant information, and as a consequence, subsequent target memory is enhanced (Egner & Hirsch, 2005). In conclusion, our results demonstrate that the specific type of control demands regulate the competition between encoding of task-relevant and task-irrelevant information which can produce opposing subsequent memory effects.

## Electronic supplementary material

Below is the link to the electronic supplementary material.

Supplementary material 1 (SAV 15 kb)

Supplementary material 2 (SAV 8 kb)

Supplementary material 3 (SAV 7 kb)

Supplementary material 4 (SAV 7 kb)

## Footnotes

1 Materials used to conduct the research (including analysis code) will be made available to other researchers for purposes of replicating the procedure or reproducing the results by email to the corresponding author.

2 The preregistered text is reported here: http://aspredicted.org/blind.php?x=vj5ue2.

3 The full text of the preregistration is reported here: http://aspredicted.org/blind.php?x=4zy67r.

## References

[CR1] Ahissar M, Hochstein S (2000). The spread of attention and learning in feature search: Effects of target distribution and task difficulty. Vision Research.

[CR2] Allport A, Styles EA, Hsieh S, Umilta C, Moscovitch M (1994). Shifting intentional set: Exploring the dynamic control of tasks. Conscious and nonconscious information processing: Attention and performance XV.

[CR3] Allport A, Wylie G, Humphreys GW, Duncan J, Treisman AM (1999). Task switching: Positive and negative priming of task-set. Attention, space and action: Studies in cognitive neuroscience.

[CR4] Anderson JR (1983). A spreading activation theory of memory. Journal of Verbal Learning and Verbal Behavior.

[CR5] Botvinick MM, Braver TS, Barch DM, Carter CS, Cohen JD (2001). Conflict monitoring and cognitive control. Psychological Review.

[CR6] Braver TS (2012). The variable nature of cognitive control: A dual mechanisms framework. Trends in Cognitive Sciences.

[CR7] Chiu YC, Egner T (2016). Distractor-relevance determines whether task-switching enhances or impairs distractor memory. Journal of Experimental Psychology: Human Perception and Performance.

[CR8] Cohen J (1988). Statistical power analysis for the behavioral sciences.

[CR9] Cohen JD, Dunbar K, McClelland JL (1990). On the control of automatic processes: A parallel distributed processing account of the Stroop effect. Psychological Review.

[CR10] Craik FIM, Klix F, Hagendorf H (1986). A functional account of age differences in memory. Human memory and cognitive capabilities: Mechanisms and performances.

[CR11] Davis H, Rosner TM, D’Angelo MC, MacLellan E, Milliken B (2019). Selective attention effects on recognition: The roles of list context and perceptual difficulty. Psychological Research.

[CR12] Dienes Z, Coulton S, Heather N (2018). Using Bayes factors to evaluate evidence for no effect: Examples from the SIPS project. Addiction.

[CR13] Dreisbach G, Wenke D (2011). The shielding function of task sets and its relaxation during task switching. Journal of Experimental Psychology: Learning, Memory, and Cognition.

[CR14] Duncan J, Dornik S (1977). Response selection rules in spatial choice reaction tasks’. Attention and performance VI.

[CR15] Egner T, Delano M, Hirsch J (2007). Separate conflict-specific cognitive control mechanisms in the human brain. NeuroImage..

[CR16] Egner T, Hirsch J (2005). Cognitive control mechanisms resolve conflict through cortical amplification of task-relevant information. Nature Neuroscience.

[CR17] Faul F, Erdfelder E, Lang A-G, Buchner A (2007). G*Power 3: A flexible statistical power analysis program for the social, behavioral, and biomedical sciences. Behavior Research Methods.

[CR18] Gratton G, Coles MGH, Donchin E (1992). Optimizing the use of information: Strategic control of activation of responses. Journal of Experimental Psychology: General.

[CR19] Jarosz AF, Wiley J (2014). What are the odds? A practical guide to computing and reporting Bayes factors. The Journal of Problem Solving.

[CR20] Jersild AT (1927). Mental set and shift. Archives of Psychology.

[CR21] Kiesel A, Wendt M, Peters A (2007). Task switching: On the origin of response congruency effects. Psychological Research.

[CR22] Krebs RM, Boehler CN, De Belder M, Egner T (2015). Neural conflict-control mechanisms improve memory for target stimuli. Cerebral Cortex.

[CR23] Lavie N, Hirst A, De Fockert JW, Viding E (2004). Load theory of selective attention and cognitive control. Journal of Experimental Psychology: General.

[CR24] Lavie N, Lin Z, Zokaei N, Thoma V (2009). The role of perceptual load in object recognition. Journal of Experimental Psychology: Human Perception and Performance.

[CR25] Los SA (1994). Procedural differences in processing intact and degraded stimuli. Memory and Cognition.

[CR26] Los SA (1999). Identifying stimuli of different perceptual categories in pure and mixed blocks of trials: Evidence for stimulus-driven switch costs. Acta Psychologica.

[CR27] Mayr U, Keele SW (2000). Changing internal constraints on action: The role of backward inhibition. Journal of Experimental Psychology: General.

[CR28] Meier B, Rey-Mermet A (2012). Beyond feature binding: Interference from episodic context binding creates the bivalency effect in task-switching. Frontiers in Psychology.

[CR29] Meier B, Woodward TS, Rey-Mermet A, Graf P (2009). The bivalency effect in task switching: General and enduring. Canadian Journal of Experimental Psychology.

[CR30] Meiran N (2000). Modeling cognitive control in task-switching. Psychological Research.

[CR31] Meiran N, Kessler Y (2008). The task rule congruency effect in task switching reflects activated long-term memory. Journal of Experimental Psychology: Human Perception and Performance.

[CR32] Milliken B, Joordens S (1996). Negative priming without overt prime selection. Canadian Journal of Experimental Psychology.

[CR33] Monsell S (2003). Task switching. Trends in Cognitive Sciences.

[CR34] Monsell S, Patterson KE, Graham A, Huges CH, Milroy R (1992). Lexical and sublexical translation from spelling to sound: Strategic anticipation of lexical status. Journal of Experimental Psychology: Learning, Memory, and Cognition.

[CR35] Muhmenthaler MC, Meier B (2019). Task switching hurts memory encoding. Experimental Psychology.

[CR36] Ortiz-Tudela J, Milliken B, Botta F, LaPointe M, Lupiañez J (2017). A cow on the prairie vs. a cow on the street: Long-term consequences of semantic conflict on episodic encoding. Psychological Research.

[CR37] Posner MI, Snyder CRR, Solso RL (1975). Attention and cognitive control. Information processing and cognition: The Loyola symposium.

[CR38] Ptok M, Thomson SJ, Humphreys KR, Watter S (2019). Congruency encoding effects on recognition memory: A stage-specific account of desirable difficulty. Frontiers in Psychology.

[CR39] Reynolds JR, Donaldson DI, Wagner AD, Braver TS (2004). Item- and task-level processes in the left inferior prefrontal cortex: Positive and negative correlates of encoding. NeuroImage.

[CR40] Richter FR, Yeung N (2012). Memory and cognitive control in task switching. Psychological Science.

[CR41] Richter FR, Yeung N (2015). Corresponding influences of top-down control on task switching and long-term memory. The Quarterly Journal of Experimental Psychology.

[CR42] Rogers RD, Monsell S (1995). Costs of a predictable switch between simple cognitive tasks. Journal of Experimental Psychology: General.

[CR43] Rosner TM, D’Angelo MC, MacLellan E, Milliken B (2015). Selective attention and recognition: Effects of congruence on episodic learning. Psychological Research.

[CR44] Rosner TM, Davis H, Milliken B (2015). Perceptual blurring and recognition memory: A desirable difficulty effect revealed. Acta Psychologica.

[CR45] Wagenmakers E-J, Verhagen AJ, Ly A, Matzke D, Steingroever H, Rouder JN, Lilienfeld SO, Waldman I (2015). The need for Bayesian hypothesis testing in psychological science. Psychological science under scrutiny: Recent challenges and proposed solutions.

[CR46] Woodward TS, Meier B, Tipper C, Graf P (2003). Bivalency is costly: Bivalent stimuli elicit cautious responding. Experimental Psychology.

[CR47] Yonelinas AP (2002). The nature of recollection and familiarity: A review of 30 years of research. Journal of Memory and Language.

[CR48] Yue CL, Castel AD, Bjork RA (2013). When disfluency is—and is not—a desirable difficulty: The influence of typeface clarity on metacognitive judgments and memory. Memory & Cognition.

